# A retrospective study of SBRT of metastases in patients with primary sarcoma

**DOI:** 10.1007/s12032-012-0256-2

**Published:** 2012-07-20

**Authors:** Christina Linder Stragliotto, Kristin Karlsson, Ingmar Lax, Eva Rutkowska, Jonas Bergh, Hans Strander, Henric Blomgren, Signe Friesland

**Affiliations:** 1 Department of Oncology, Radiumhemmet, Karolinska University Hospital, 171 76 Stockholm, Sweden; 2 Department of Medical Physics, Radiumhemmet, Karolinska University Hospital, Stockholm, Sweden; 3 Directorate of Medical Imaging and Radiotherapy, School of Health Sciences, University of Liverpool, Liverpool, UK

**Keywords:** Stereotactic body radiotherapy, Sarcoma, Metastases, High-dose fraction, Body-frame

## Abstract

We retrospectively reviewed the results of stereotactic body radiotherapy (SBRT) in 46 patients with a total of 136 metastases from primary sarcoma. The purpose of this study was to evaluate the overall response rate and side effects of SBRT in metastatic sarcoma. The patients were treated at Karolinska University Hospital between 1994 and 2005, using 3D conformal multifield technique and a stereotactic body-frame. Prescribed doses ranged from 4 to 20 Gy per fraction in 1–5 fractions, with total doses of 10–48 Gy. All 46 patients were diagnosed with a primary sarcoma. The treated metastases were localized mainly in the lungs. A total number of 136 metastases were treated (1–14 per patient). Overall response rate (local control = CR, PR and SD) for each tumour was 88 % (119/135). Median follow-up was 21.8 months (range 2.7–112.8 months). Thirteen patients (31 %) were long-term survivors (>36 months), and 5 patients are still alive after last follow-up. Two cases of serious non-lethal side effects were seen, one patient had a colon perforation and another patient had contracture of the hip region. SBRT is a safe, convenient and effective non-invasive treatment with high local control for patients with metastatic sarcoma.

**Electronic supplementary material:**

The online version of this article (doi:10.1007/s12032-012-0256-2) contains supplementary material, which is available to authorized users.

## Introduction

Since the 1980s, intracranial stereotactic radiation techniques have been used [1, 2]. The earliest publications on radiation therapy of extracranial targets involving stereotactic positioning are from Lax [3], who developed a stereotactic body-frame for exact tumour localization and reproducible fixation and treatment with an accelerator. Since then this method has been used continuously at our centre for various types of metastases. Several studies of stereotactic body radiation therapy (SBRT) now show a high local control rate for a large number of tumour types, both primary tumours and metastases [4–8]. In last years, several studies have also shown the effectiveness of SBRT of primary lung carcinoma.

Sarcomas are a heterogeneous group of rare mesenchymal tumours (approximate incidence of 337 per year in Sweden) [9]. Multimodality treatment often provides good local control when the tumour is located in an extremity. Neoadjuvant chemotherapy is useful for osteosarcoma. However, for soft tissue sarcoma, a few chemotherapeutic regimen—mostly adriamycin, cyclophosphamide and ifosfamide based—yield responses in about 20 % of cases. Most centres offer a combination of these drugs for adjuvant treatment of aggressive soft tissue tumours. There is a lack of data supporting the use of neoadjuvant therapy in soft tissue sarcomas. Soft tissue sarcomas are also treated with preoperative or postoperative radiation, the ideal sequence of treatment still remains controversial [10, 11]. Advanced-stage bone and soft tissue sarcoma still represent a major challenge, because in spite of aggressive treatment, the disease-free progression is <10 % at 5 years [12]. Until now, the choice of therapy for single metastases has been surgery. Several data exist on improvement of overall survival after resection of residual metastatic disease [13–16]. All patients with metastatic disease should therefore be carefully evaluated for possible resection. To our knowledge, there is only one report showing the efficacy of SBRT in lung metastases from primary sarcoma [17].

Surgery is, thus, still the therapy of choice for treatment of single sarcoma metastases. However, all patients are not fit for surgical treatment because of different reasons, and there is an urgent need for alternative therapy options. Radiotherapy given with conventional technique is sometimes used for palliative treatment, but the doses given per fraction are often considered too low to achieve adequate local control, and the results are disappointing. Radiotherapy given by SBRT when hypofractionated doses are delivered within a short period of time can provide curable doses and possibility to high local control. This retrospective study gives the experience from Karolinska of SBRT for patients with metastatic sarcoma.

## Materials and methods

### Patients

We retrospectively reviewed the results of SBRT of 46 patients with non-resectable metastases from primary sarcoma. The patients were treated at Karolinska University Hospital between September 1994 and October 2005. The study has been approved by a local ethics committee.

All patients with histologically confirmed primary sarcoma who were treated with SBRT were included in the study. There were 29 females and 17 males with a mean age of 47.9 years (median 52.3 years, range 8.7–83.0 years) at treatment start. Five of the patients were children (<18 years at treatment start). A total number of 136 lesions were treated (range 1–14 per patient); five of these were re-irradiated tumours. One patient had two different re-irradiated tumours. The tumours were treated at 1–5 occasions per patient; one occasion, therefore, implies that more that one metastasis could be treated during the same SBRT session.

The dose was generally prescribed to about 65 % isodose and varied between 4 and 20 Gy per fraction in 1–5 fractions with total doses of 10–48 Gy. The prescribed dose expressed in equivalent dose in 2 Gy fractions (EQD_2_) ranged between 26.0 and 162.0 Gy with α/β-value 3 Gy. All patients but one had metastatic disease and were medically inoperable, mainly because of a considered poor general condition and an advanced disease with multiple metastases. Most patients underwent SBRT after their disease progressed despite chemotherapy. For some patients, SBRT was applied to new metastases that developed after the first SBRT. The median time of follow-up was 21.8 months (mean 32.5 months, range 2.7–112.8 months). Table 1 gives the patient and tumour characteristics.

**Table 1:** Patient and tumour characteristics

Characteristics	
*Gender*	
Male	17
Female	29
*Age* ^a^ *(years)*	
Mean	47.9
Median	52.3
Range	8.7–83.0
*CTV (cm* ^*3*^ *)*	
Mean	62.9
Median	11.4
Range	0.0–864.2^b^
*Histology* (46 patients)	
Soft tissue sarcoma^c^	28
Uterine sarcoma	7
Osteosarcoma	5
Ewing/PNET^d^	5
Nerve-derivated sarcoma	1
*Localization* (136 tumours)	
Lung	97
Liver	14
Abdomen/pelvis^e^	12
Pubic bone	3
Thorax	2^f^
Gluteus	2
Femur	2
Psoas	1^f^
Other^g^	3

^a^If a patient was treated at several times, the age at the first treatment is presented here

^b^A volume of 0.0 cm^3^ implies that the volume was <0.05 cm^3^

^c^Leiomyosarcoma (*n* = 12), synovial sarcoma, (*n* = 4), liposarcoma (*n* = 4), malignant fibrous histocytoma (MFH) (*n* = 4), angiosarcoma (*n* = 1), fibrosarcoma (*n* = 1), pleomorf sarcoma (*n* = 1) and unspecified soft tissue sarcoma (*n* = 1)

^d^Ewing (*n* = 3), primary neuroectodermal tumour (PNET) (*n* = 1), Ewing/PNET (*n* = 1)

^e^Abdomen (*n* = 5), pelvis (*n* = 5) and located in both abdomen and pelvis (*n* = 2)

^f^Location of the three primary tumours (in three patients) was in thorax, psoas and in the trapezius muscle

^g^Suprarenal gland (*n* = 1), trapezius muscle (*n* = 1) and around a thoracal-lumbal vertebrae body (*n* = 1)

### Stereotactic body radiotherapy (SBRT)

The methodology of SBRT and the stereotactic body-frame (Electa Oncology Systems) has previously been described [3, 18]. The body-frame is used for immobilization of the patient, for accurate geometrical localization and verification of the target and for set-up in the treatment room. The patient is reproducibly fixed in the frame by a custom-fitted vacuum pillow. On the inner walls of the frame indicators visible at CT are mounted, these indicators define the stereotactic system. There are scales mounted on the outside of the frame for set-up according to the lasers, indicating the isocentre coordinates in the treatment room, and these scales correlate with the inner wall indicators seen on CT.

To minimize tumour movements due to breathing, an abdominal pressure device was used for patients with diaphragm movements larger than 10 mm in the cranio-caudal direction, checked by fluoroscopy. A first CT examination was done with the patient fixed in the frame before the dose planning, and another CT examination was done shortly before the start of the first fraction. The two CT studies were matched with each other to study the reproducibility of repositioning the target in the stereotactic system. The repositioning reproducibility of tumours in the stereotactic system was generally within 6–7 mm in the transverse plane and within 10 mm in the cranio-caudal plane [18]. Generally, the thickness of the CT slices was 5 mm.

CTV was defined on the CT images, and in most cases, CTV was identical to the gross tumour volume (GTV). To the CTV, a margin of 5–10 mm was added in the transverse plane, and a margin of 10 mm was added in the cranio-caudal direction to obtain planning target volume (PTV). These margins include set-up and repositioning inaccuracy and internal motions of the target [18].

### Treatment technique and dose distribution

The treatment of the patients in this study was a conformal 3D technique with 5–8 coplanar or non-coplanar static beams [3, 18] with photon energy 6 MV. The directions of the beams were spread in a large solid angle, taking into account the localization of organs at risk [19]. The beams were shaped by multi-leaf collimators (MLC) to conform to the target.

An inhomogeneous dose distribution within the PTV was used [3, 20]. The dose was generally prescribed to the 65 % isodose at the periphery of PTV. In the central parts of the PTV, the dose was about 50 % higher than the prescribed dose. The benefit of this inhomogeneous dose distribution inside the PTV is that the central parts might contain more hypoxic cells with lower radiosensitivity than the periphery [3, 19, 20]. For each patient, an individualized three-dimensional dose plan (TMS, Helax) was made using a pencil beam algorithm.

### Geometrical verification

An important part of SBRT is the use of CT for direct geometrical verification of the target position in the stereotactic system, instead of the indirect verification of bony structures done by portal imaging [18]. Because of that, a second CT examination was done before the first treatment to verify the reproducibility of the position of the target in the stereotactic system. If this showed reproducibility within the margin between CTV and PTV, no further geometrical verification imaging was done.

### Fractionation and fractionation sensitivity of sarcoma

In this present study, 4–20 Gy in each fraction was prescribed to the periphery of the PTV. The number of fractions varied between 1 and 5, generally given every second day. There are several factors to consider when deciding the dose per fraction, the number of fractions and the time interval between the irradiations. Such factors can be the size of CTV, the dose distribution in the target and surrounding radiation-sensitive normal tissues [19]. In Table 2, the frequency of different fractionation schedules in this study can be seen.

**Table 2:** Treatment characteristics for all tumours

Total dose (Gy)	No of fractions	Prescribed dose EQD_2_ (Gy)	Mean dose to CTV EQD_2_ (Gy) range	No of tumours
10	1	26.0	48.8	1 (0.7 %)
20	1	92.0	204.3–205.5	2 (1.5 %)
16	2	35.2	64.2	1 (0.7 %)
20	2	52.0	85.0–227.3	8 (5.9 %)
30	2	108.0	159.7–314.7	35 (25.7 %)
21	3	42.0	61.8–67.2	2 (1.5 %)
24	3	52.8	83.7	1 (0.7 %)
30	3	78.0	84.4–167.2	9 (6.6 %)
36	3	108.0	189.6	1 (0.7 %)
45	3	162.0	237.0–402.8	25 (18.4 %)
24	4	43.2	54.2	1 (0.7 %)
28	4	56.0	105.2	1 (0.7 %)
32	4	70.4	115.3–122.7	2 (1.5 %)
36	4	86.4	115.1	1 (0.7 %)
40	4	104.0	155.3–225.7	21 (15.4 %)
48	4	144.0	198.7	1 (0.7 %)
20	5	28.0	39.0–40.5	2 (1.5 %)
25	5	40.0	53.3	1 (0.7 %)
30	5	54.0	83.2–101.5	5 (3.7 %)
35	5	70.0	115.6	1 (0.7%)
40	5	88.0	101.1–153.4	15 (11.0 %)

Data of fractionation sensitivity for sarcoma are sparse. However, it is reported that some sarcoma types have very low α/β ratios [21]. For example, Gagnon et al. [22] reported about an α/β value of approximately 1 Gy for osteosarcoma, and Thames and Suit [23] reported an α/β ratio of 0.4 Gy (95 % confidence interval: −1.4, 5.4) for liposarcoma. Because of complexity in fractionation sensitivity for different kinds of sarcoma, the generally used α/β ratio of 3 Gy for late-responding tissue [24] has been used for all tumours in this study.

For purposes of comparison of the different fractionation schedules, the EQD_2_ was calculated assuming an α/β ratio of 3 Gy. The results are shown in Table 2 for the prescribed dose and the mean dose to the CTV. Information about fractionation for one patient with two tumours treated at two different sessions was not available. For one tumour in another patient, the dose plan, with dose distribution and DVH, was not available.

### Follow-up and definition of responses

At the time of treatment, all patients had one or more known lesions that had increased in size as assessed by repeated CT scans of the thoracic cavity and abdomen. For therapy evaluation, a first CT scan after treatment was done after a period of 2 months to 1 year. Due to the retrospective data collection, no fixed evaluation time was determined. Response is therefore based on CT scans performed in a period of 1.2–32.7 months (mean 7.1 months, median 4.6 months) and should therefore be regarded as best response. If the patient had more than one CT scan, the one closest to 6 months after treatment was evaluated. However, in most cases, there were no differences in response seen in CT scans done later than 6 months after treatment. In this material, the patients rather progressed with development of new metastases than progression of the SBRT-treated lesions. Sometimes it was difficult to evaluate the response on the CT scans due to development of fibrosis and scar tissue after treatment. Eight tumours (4 patients) were not eligible for evaluation of time to best response. Furthermore, some of the patients came from abroad or were followed at other hospitals and were not followed regularly at our hospital after SBRT.

In most cases, it was not possible to get a histological confirmation of the metastases. Instead, CT, conventional radiography or PET was used. An increase in the size of the lesion between the two latest CT scans at least 2 months apart and a malignant appearance assigned the lesions as suspected metastases. However, open biopsies or needle aspiration for histopathological examinations were obtained from patients with a long duration between primary treatment and development of recurrent disease.

For the radiological evaluation after SBRT, the response was considered complete (CR) if there was no visible tumour, partial (PR) if the cross-sectional tumour diameter was reduced by at least 50 % and stable disease (SD) if there was less than 50 % decrease or less than 25 % increase in this parameter. Local failure or progressive disease (PD) was defined as over 25 % increase in cross-sectional tumour diameter.

### Statistical methods

Relations between variables were tested with Spearman correlation. Survival analysis was performed with the Kaplan–Meier method and the life table technique using the Wilcoxon (Gehan) exact test comparing differences in survival between groups. Multivariate analysis was performed with Cox regression analysis. Background factors to survival were response, mean dose and CTV volume.

## Results

### Local control and response

Of the 136 tumours treated in this study, 39 (29 %) showed CR, 27 (20 %) PR, 53 (39 %) SD and 16 (12 %) PD as best response. For one tumour, information about response was not available. The overall response rate (local control = CR, PR and SD) for all evaluable tumours was 88 % (119/135).

There were variations in responses, but no obvious differences between different histological types of sarcoma (Table 3). However, 12 out of 17 (71 %) uterine sarcomas responded with CR, but no significant difference in survival for this group could be seen. The variations in tumour response between different sarcoma types raised the question about differences in fractionation for different histological types for the tumours in this study. However, this was explored with futile results.

**Table 3:** Distribution of best response for different kinds of sarcoma histology

	CR	PR	SD	PD	na
Soft tissue sarcoma^a^ (*n* = 88)	19 (22 %)	17 (19%)	42 (48 %)	9 (10 %)	1 (1 %)
Uterine sarcoma (*n* = 17)	12 (71 %)	4 (24%)	–	1 (6 %)	–
Osteosarcoma (*n* = 16)	3 (19 %)	–	9 (56 %)	4 (25 %)	–
Ewing/PNET^b^ (*n* = 14)	4 (29 %)	6 (43%)	2 (14 %)	2 (14 %)	–
Nerve-derivated sarcoma (*n* = 1)	1 (100 %)	–	–	–	–

^a^Leiomyosarcoma, synovial sarcoma, liposarcoma, malignant fibrous histocytoma (MFH), angiosarcoma, fibrosarcoma, pleomorf sarcoma and unspecified soft tissue sarcoma

^b^Primary neuroectodermal tumour (PNET)

When analysing each treated tumour separately, the fact that some of these are partly coupled since they were located in the same patient must be taken into consideration. Thirty-one (67 %) of the patients in this study were treated for more than one tumour and were therefore represented several times in the analysis of each tumour separately.

**Fig. 1:**
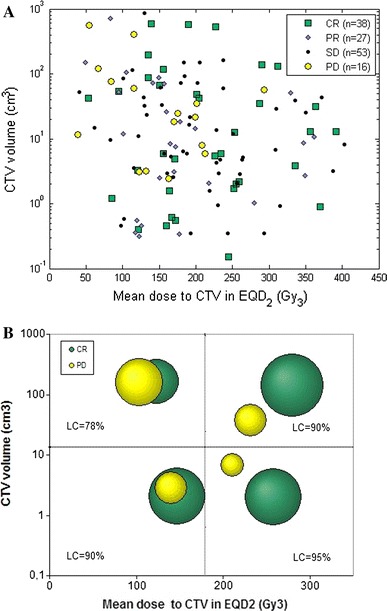
**a** CTV volume and mean dose to CTV in EQD_2_ for tumour with best response CR, PR, SD, PD, respectively. For one tumour with complete response dose, information was not available. **b** The points in **a** are divided into four subgroups: smaller/greater than median CTV and smaller/greater than median EQD2, illustrated by the *dashed lines*. The area of the *circles* represents the percentage of CR, respectively, PD in each subgroup. Local control (LC) for each subgroup is also given

Figure 1 shows the response of the tumours as a function of the volumes and the mean doses to the CTVs. The mean CTV volume was 62.9 cm^3^ (median 11.4 cm^3^, range 0.0–864.2 cm^3^). In Fig. 1a, showing all tumours separately, it can be seen that for tumours with PD, there was a tendency of larger CTV volume and lower mean dose to CTV than for tumours with local control (CR, PR and SD); this is shown more in detail in Table 4. In Fig. 1b, the CR and PD tumours are grouped according to CTV volume and mean dose. This shows that tumours with response PD more frequently had larger CTV volumes and lower mean doses. This is in agreement with local control (LC = 78 %) being lowest in this group. For tumours with response CR, there was no clear correlation with the response to CTV volume or mean dose to CTV. This is further elaborated in the Discussion part.

**Table 4:** Mean dose to CTV expressed in EQD_2_ and CTV volume, for all tumours and tumours stratified according to best response

	Mean dose in EQD_2_ (Gy)	CTV (cm^3^)
*All tumours* ^a^ (*n* = 134/136)		
Mean (std)	194.5 (84.5)	60.2 (138.3)
Median (range)	181.1 (39.0–402.8)	11.4 (0.0–864.2)
*Tumours with best response CR* ^a^ (*n* = 38/39)		
Mean (std)	213.0 (83.6)	72.9 (153.6)
Median (range)	204.4 (53.3–392.1)	5.9 (0.0–594.1)
*Tumours with best response PR* (*n* = 27)		
Mean (std)	174.4 (86.7)	55.5 (137.0)
Median (range)	156.7 (48.8–361.3)	10.6 (0.3–711.7)
*Tumours with best response SD* (*n* = 53)		
Mean (std)	205.8 (83.1)	45.1 (121.8)
Median (range)	192.6 (40.5–402.8)	9.5 (0.0–864.2)
*Tumours with best response PD* (*n* = 16)		
Mean (std)	147.1 (68.4)	88.0 (159.5)
Median (range)	147.5 (39.0–293.7)	23.0 (2.4–559.7)

^a^For one tumour, information about response was not available; for another (responding with CR), information about fractionation schedule was not available and mean dose could not be calculated

Best response was significantly correlated with mean dose to CTV in EQD_2_ (*p* = 0.018), with a non-parametric correlation factor for Spearman’s ρ of 0.204, if tumours responding with PR and SD were grouped together. For the volume of CTV, no significant correlation with the response was found (*p* = 0.149, Spearman’s ρ = −0.125).

### Survival

**Fig. 2:**
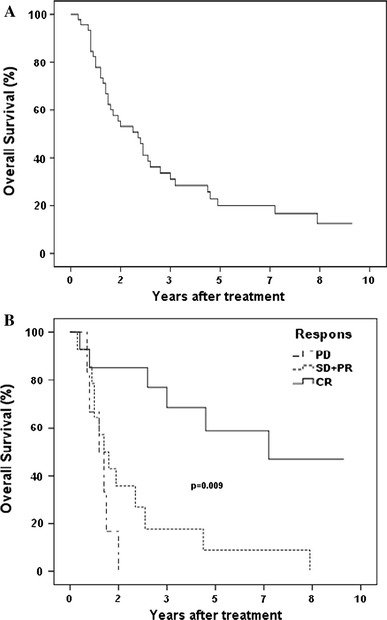
**a** Kaplan–Meier plot of total survival in months after first stereotactic treatment. 5 patients are still alive, 36 patients have died and 5 patients are lost for follow-up. **b**. Kaplan–Meier plot of survival in months after first stereotactic treatment for each patient grouped according to best response for all the patients’ tumours, and only patients with tumours responding the same way are presented here

The median overall survival time for the patients in this study was 26.3 months; a Kaplan–Meier plot for overall survival is shown in Fig. 2a. The 2-year overall survival was 50.7%, the 3-year overall survival was 33.6 % and the 5-year overall survival was 20.0 %. Currently, 5 patients are alive, 36 patients have died and 5 patients are lost for follow-up. Thirteen of 42 patients (31 %) are long-term survivors, that is, survived longer than 36 months after first SBRT. Four patients could not be evaluated for long-term survival since they were lost for follow-up before 36 months.

For the different histology types of sarcoma, the median (range) survival for soft tissue sarcoma (22 patients) was 14.2 months (2.7–98.5), for uterine sarcoma (5 patients) 18.1 months (9.2–31.4), osteosarcoma (4 patients) 17.7 months (9.9–24.4), Ewing/PNET (4 patients) 13.6 months (3.4–35.6) and for nerve-derivated sarcoma (1 patient), the survival was 55.8 months.

To evaluate the correlation between tumour response and survival, the patients were stratified into groups with same response of all tumours, excluding patients with different response for different tumours. The median survival time for patients with tumours responding with CR was 81.7 months, for patients with PR and SD grouped together, the median survival time was 15.0 months and for patients with PD, the median survival was 13.0 months. The Kaplan–Meier survival functions for these groups are shown in Fig. 2b.

Wilcoxon (Gehan) statistics for overall Kaplan–Meier survival curves for patients stratified according to the response of the tumour(s), excluding patients with several tumours with mixed response, showed that the disagreement between the three survival functions is highly significant (*p* = 0.009). This indicates that better response significantly correlates with longer survival. Because of the difficulty of evaluating whether the response was PR or SD from the CT images, tumours with these responses were grouped together. Often, fibrosis can be seen around the treated lesions and can sometimes be hard to differentiate from tumour tissue.

The analysis of survival for patients grouped according to the size of the largest CTV showed a significant correlation (*p* = 0.037); a smaller CTV correlated with a better survival (correlation factor 0.002). This indicates the importance of a higher total dose for larger tumours.

The survival was also evaluated according to the number of treated tumours in the patient, but no significant correlation was found (*p* = 0.558, correlation factor −0.039).

### Side effects of SBRT

Out of the 46 patients in the study, it was possible to evaluate side effects for 34 patients. Twenty-three of these (68 %) patients developed at least one side effect after treatment with SBRT. In the remaining 13 patients, it was not possible to evaluate side effects retrospectively. Two cases of serious side effects were seen in this study: one perforation of the colon and one contracture of the hip. In neither of these cases, the side effect was lethal. In all other cases, side effects were mild to moderate; the most common side effects were cough and dyspnoea (Table 5).

**Table 5:** Number of side effects in evaluable patients

Side effects	Number of patients^a^
No side effects	11
Cough	8
Dyspnoea	7
Fatigue	4
Pleural exudates	4
Skin rash	2
Thoracic pain/pain in the ribs	2
Abdominal pain	1
Colon perforation	1
Contracture of the hip	1

^a^One patient could have more than one side effect

## Discussion

This retrospective study of the first 46 patients with inoperable metastasizing sarcoma shows an 88 % rate of local disease control as best response. This compares favourably with other tumours treated with SBRT. For example, renal cell carcinoma and inoperable non-small cell lung cancer had 90–98 % [25] and 88 % [26] local control, respectively. Our results compare well even with results achieved when intracerebral metastases from various primary tumours were treated with stereotactic radiation therapy or gamma-knife, which result in a local control of about 90 % [1, 2]. Thus, this study shows that even sarcomas, traditionally considered as radioresistant tumours, like renal cell carcinoma or non-small cell lung cancer can respond well to SBRT.

**Fig. 3:**
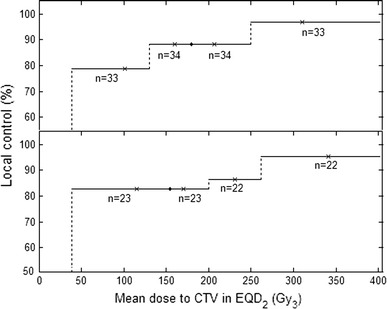
Local control (CR, PR or SD) as best response versus mean dose to CTV in EQD_2_. Tumours were grouped according to mean dose to CTV in EQD_2_ into four groups with about equally number of tumours. The upper graph contains all but two (missing information) tumours. The lower graph includes all but one (missing information) tumours with a diameter between 1 and 5 cm (volume 0.52–65.45 cm^3^), excluding tumours with large, respectively, small volumes

An important question is whether a dose–response relation can be deduced from this study. For this purpose, the tumours were grouped into four intervals of different mean dose in EQD_2_ delivered to the CTV. Figure 3, upper panel, shows local control (CR + PR + SD) versus EQD2 for the four groups. The mean volume of the CTV in these four groups was 85.1 cm^3^ (group with lowest dose), 73.5, 60.6 and 21.1 cm^3^ (group with highest dose), respectively. The statistical analysis showed a significant (*p* = 0.023) association between local control and mean dose to CTV in EQD_2_, but not between local control and the volume of CTV (*p* = 0.088). However, due to a possible confounding influence on the dose–response relationship, by a correlation between mean dose to the CTV and the volume of the CTV, tumours smaller than 1 cm (spherical volume of 0.52 cm^3^) and larger than 5 cm (spherical volume of 65.45 cm^3^) were excluded in the analysis (Fig. 3, lower panel). For this subgroup, local control was calculated in another four groups according to mean dose in EQD_2_. The mean CTV volumes in these groups were 23.1, 9.8, 12.5 and 17.8 cm^3^, respectively. The data given in Fig. 3 imply that a mean dose to CTV in the order of 20 Gy/fraction given in three fractions is needed to achieve a local control in the order of 90 %.

The equivalent uniform dose (EUD) [27] was calculated for CTV of each tumour in order to see whether it was a descriptor for local control, see electronic supplement.

There has been a long learning period; different doses were given to different tumour locations. This was mostly due to the possible risk of complications for adjacent tissues and lack of literature data from SBRT at that time. In this study, however, all patients were treated at one site by only two physicians and built up a basis for substantial continuity and reproducibility and possibility to proceed with clinical trials to introduce SBRT as an alternative therapy option.

The majority of our patients were treated for metastases in the lungs. However, treating tumours in other anatomical sites such as liver, adrenal gland and retroperitoneum is technically possible. The limitation is often the proximity to organs at risk like medulla, main bronchus, ventricle, etc.

Several patients were retreated with SBRT when they developed new metastases at other locations. One patient was treated with SBRT for a total of 14 different metastases. It is interesting that the side effects still were very mild. Thus, the aim of regular CT scans was also to detect new metastases at an early stage and treat them as soon as possible with SBRT. Several studies [13, 15] show that a subset of patients can be cured when a complete response of metastatic disease is achieved either by surgery alone or in a combination of surgery and chemotherapy. Our study suggests that SBRT has an important role to improve overall survival in patients with metastatic disease. In one abstract [17], the survival at 2.5 years was 73 %. In our study, the survival was 39 % at 2.5 years. The difference could be due to different selection criteria between the patient materials. Almost all patients in our material had more than one metastatic lesion before treatment with SBRT.

Until now, there are no studies comparing the efficacy of SBRT and surgery. When patients had multiple metastases, CT was used to detect the most rapidly growing ones, and these were treated with SBRT to avoid pain or compression symptoms from metastases. Our results indicate that patients with small tumours benefited more from this treatment than patients with larger tumours. Six patients with metastatic disease were even cured. Therefore, it could be of value to examine patients with CT scans. We now follow our patients regularly every third month during the first 2 years and then every sixth month. In several patients, the tumour diameter remained unchanged during a long observation period. This could be due to an inhibition of tumour cell growth or a substitution of tumour by fibrosis. In PR or SD, tumour cells could still be present with potential to grow with time. Reduction in volume may not be an optimal marker for evaluating response. There is great difficulty in evaluating response on CT scans due to the development of fibrosis around the irradiated area. In the future, PET/CT may help in response evaluation after SBRT and add valuable information to routinely done CT scans for better planning of therapy.

The side effects in this retrospective analysis were mild and the most common ones were cough, dyspnoea and pleural effusion. All these symptoms could also be due to tumour progression. It is therefore impossible to evaluate the side effects adequately in this retrospective study with proper toxicity grading. Two cases of serious side effects were seen, one patient with a colon perforation and another patient with a contracture of the hip. The patient with the colon perforation was treated with 12 Gy × 3 to a target with a CTV volume of 576 cm^3^ with the colon partly included in the full dose region. The patient with contracture of the hip was treated twice, first with 10 Gy × 4 to target with a CTV volume of 73 cm^3^ and 1.5 years later with 9 Gy × 4 to a target of 403 cm^3^. Both these targets were located close to the hip, with parts of the hip within the full dose region. Both patients were treated over 10 years ago and would probably not be given the same doses today.

Since this is a retrospective study, there is no systematic follow-up or proper response evaluation at given time points available. However, the side effects of the treatment were mild, the local response was comparable with surgical intervention and a long-term survival (>36 months) of 30 % is remarkable for metastasising sarcoma, compared to around 70% 5-year overall survival in non-metastasizing sarcoma [28]. This demonstrates the usefulness of SBRT in metastatic sarcoma and the urgent need for further development of the method and evaluation within controlled clinical trials in future.

We conclude that SBRT is a safe, convenient and effective non-invasive treatment for high local tumour control for patients with metastatic sarcoma.

## Electronic supplementary material

Below is the link to the electronic supplementary material.

Supplementary material 1 (PDF 64 kb)
