# Supplementary material for: A retrospective study of SBRT of metastases in patients with primary sarcoma
Source: Med Oncol. 2012 Jul 20;29(5):3431–9. doi: 10.1007/s12032-012-0256-2 (PMC3505552; doi:10.1007/s12032-012-0256-2)
Supplement: Supplementary file 1 — Supplementary material 1 (PDF 64 kb) [file 12032_2012_256_MOESM1_ESM.pdf]

Electronic supplement – *Equivalent uniform dose*

The equivalent uniform dose (EUD) [1] was calculated for CTV of each tumour in order to investigate how well it predicts local control. EUD is the dose in Gray, given homogeneously to a volume of interest, which predicts the same number of surviving clonogens as the actual dose distribution. It reduces an inhomogeneous dose distribution in the target to a single dose value, given homogeneously to the target, which causes the same effect on the tumour.

The concept of EUD is related to the tumour control probability (TCP), since an increase in the EUD reduces the number of surviving cells in the target. If the EUD is higher than the target prescription dose, the expected TCP is higher than for the prescription dose given homogeneously to the target. Close to organs at risk the dose is often reduced in the target, and in some cases the EUD falls below the prescription dose. This means that it has not been possible to give the target the dose believed necessary for local tumour control. Thus a low EUD is expected to correlate with local failure.

In this study, the EUD to the CTV (generally equal to GTV) was calculated, in order to evaluate the impact of dose below the prescription dose to parts of the macroscopic tumour on risk for progressive disease.

Since the fractionation schedules in this study varied we chose to normalise the EUD to 2 Gy fractions to be able to compare them with the prescribed doses in EQD_2. When choosing the reference dose in the EUD formula to be 2 Gy the corresponding survival fraction is assumed to be 0.5 [2].

For 27 tumours (16 patients) the EUD to the CTV was lower than the prescribed dose in EQD_2, see Figure 4. For five (19%) of these the best response was CR, 4 metastases (15%) responded with PR, 12 (44%) had SD and 6 metastases (22%) had PD as best response. Thus these tumours had a higher frequency of PD compared with the 106 tumours with EUD higher than the prescribed dose, out of which 10 (9%) responded with PD. Nine of the metastases with EUD lower than the prescribed dose were in three children (<18 years at treatment); the best response for these metastases were 2 CR, 6 SD and 1 PD.

Tumours treated with EUD higher than the prescribed dose had a median CTV volume of 6.0 cm^3 (mean 40.2, range 0.0-575.8) while tumours with EUD lower than the prescribed dose had a median CTV volume of 53.2 cm^3 (mean 114.5, range 0.6-864.22). Thus, tumours with EUD lower than the prescribed dose generally have larger volumes and higher local failure frequency.

The results of the EUD calculations, shown in Figure 4, illustrate the importance of adequate dose-coverage of the complete CTV to obtain a high incidence of local control. Interestingly, relatively high figures of local control were also seen for the two groups with the lowest mean dose to the CTV, however, as shown in Figure 1b.

References

[1] Niemierko, A. Reporting and Analyzing Dose Distributions: A concept of equivalent uniform dose. Med Phys 1997;24:103-110.

[2] Schefter, T, Kavanagh, B, Timmerman, R, Cardenes, H, Baron, A and Gaspar, L. A phase I trial of stereotactic body radiation therapy (SBRT) for liver metastases. Int J Radiat Oncol Biol Phys 2005;62:1371-1378.

Figure 4

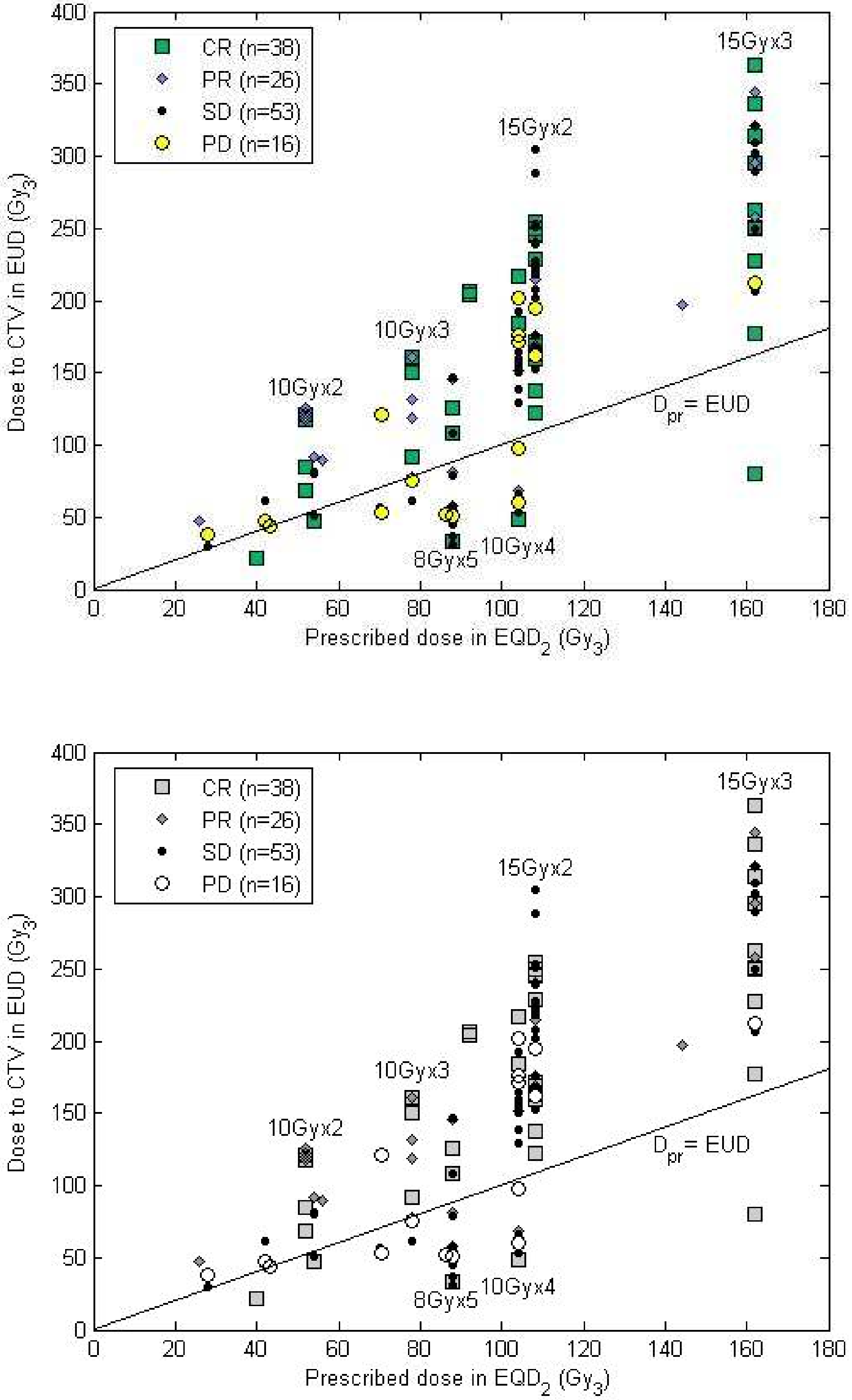

Fig. 4. Prescribed dose to PTV in EQD_2 versus delivered dose to CTV in EUD normalised to 2 Gy fractions. For three tumours information about EUD was not available. D_{pr} = prescribed total dose in EQD_2.
